# The OPAT opportunity for beta-lactam individualization

**DOI:** 10.1017/ash.2024.367

**Published:** 2024-09-04

**Authors:** Lindsey M. Childs-Kean, Christina G. Rivera, Veena Venugopalan, Madelyn J. Johnson, Erin F. Barreto

**Affiliations:** 1 Department of Pharmacy Education and Practice, University of Florida College of Pharmacy, Gainesville, FL, USA; 2 Department of Pharmacy, Mayo Clinic, Rochester, MN, USA; 3 North Dakota State University College of Pharmacy, Fargo, ND, USA

## Abstract

Beta-lactam therapeutic drug monitoring has been growing in prevalence in the acute care hospital setting. Expansion of its use to outpatient parenteral antimicrobial therapy requires careful consideration of potential logistical and therapeutic barriers.

## Introduction

Beta-lactam antibiotics are the backbone of the treatment of all major infectious syndromes in acutely ill patients. Estimates indicate that up to 70% of hospitalized patients treated with an antibiotic receive a beta-lactam.^1^ As hydrophilic small molecules, changes to volumes of distribution with resuscitation, altered host physiology, and dynamic end organ function each contribute to substantial variability in observed beta-lactam drug concentrations.^2^ As time-dependent antimicrobials, subtherapeutic beta-lactam levels can decrease clinical and microbiologic cure and contribute to development of antimicrobial resistance.^2^ In rare circumstances, supratherapeutic levels can increase the risk for dose-dependent adverse effects such as neurotoxicity.^3^

Therapeutic drug monitoring (TDM) is one strategy used to individualize doses, thereby improving effectiveness and safety. Use of beta-lactam TDM to individualize therapy is increasing in popularity worldwide, albeit only used rarely in the United States.^4^ Data on the benefits of TDM-guided beta-lactam dose optimization are promising, but at times conflicting. A meta-analysis of TDM-guided beta-lactam dosing in critically ill patients revealed 85% higher target attainment, 17% greater clinical cure, 14% greater microbiological cure, and 21% reduction in treatment failure.^5^ Despite improved pharmacokinetic/pharmacodynamic (PK/PD) target attainment, recently conducted randomized controlled trials have failed to show improved clinical outcomes or decreased mortality.^6–8^ Some study limitations that may have contributed to reduced observed benefit of beta-lactam TDM include delays in time to sampling, heterogeneous patient populations, and variability in PK/PD targets. Generally, three different PK/PD targets have been variably tested for beta-lactams to optimize clinical effectiveness: 40–60% ƒT > MIC, 100% ƒT > MIC, and 100% ƒT > 4xMIC, making comparisons across studies challenging.^3^ Safety thresholds for beta-lactams are less clear. There does appear to be an exposure response relationship between beta-lactams and neurotoxicity and cytopenias.^3,9^ Other adverse effects appear more idiosyncratic (e.g., nephrotoxicity and hepatotoxicity). TDM may be useful to minimize beta-lactam associated toxicities, but these are relatively rare and hard to systematically evaluate. The majority of evidence describing the impact and implementation of beta-lactam TDM is based in the acute hospital setting. Beta-lactam antibiotics are also used in the outpatient setting, including as part of outpatient parenteral antimicrobial therapy (OPAT) programs.

OPAT, defined as the administration of parenteral antimicrobial therapy for at least 2 doses on different days without an intervening hospitalization, has been shown to be clinically efficient and cost-effective.^10^ Nearly 250,000 patients in the US receive OPAT annually.^10^ Prolonged courses of antibiotics over weeks to months predominate in OPAT due to difficult to treat sources of infection (e.g. bone/joint infections and endocarditis) and/or resistant organisms. The prolonged exposure to antibiotics can lead to more opportunities for adverse events (AEs). Approximately 20–30% of patients treated with OPAT experience at least one AE.^11,12^ To optimize the pharmacokinetics and pharmacodynamics of treatment regimens and decrease the risk for AEs, TDM may be considered.

TDM in OPAT is commonplace for vancomycin or aminoglycosides, but rare for beta-lactam antibiotics due to clinical and logistical nuances. At our centers, beta-lactam TDM has been used in select patients involved with the OPAT program. This concise communication aimed to illustrate the potential of beta-lactam TDM in OPAT through a patient case, characterize key challenges and opportunities associated with beta-lactam TDM in OPAT, and provide a framework for others who aim to implement beta-lactam TDM in the OPAT setting.

## Clinical application

To illustrate the potential for beta-lactam TDM in the OPAT setting, we first describe the case of an individual with suspected dose-dependent beta-lactam toxicity who benefited from TDM:

A 76-year-old man needed OPAT with piperacillin/tazobactam for a *Pseudomonas aeruginosa* lung abscess. The initially planned three-week course was prolonged to two months based on laboratory features, imaging, and clinical signs and symptoms. During therapy, the patient experienced new-onset confusion which prompted an emergency department visit and neurologic workup. Laboratory and imaging findings, including a head CT, were unrevealing. The patient was dismissed back to the care of the OPAT team who ordered a piperacillin/tazobactam concentration. At the time of the drug concentration, the patient was treated with 13.5 grams/day of piperacillin/tazobactam via continuous intravenous infusion (weight 41 kg, serum creatinine 0.6 mg/dL, estimated creatinine clearance 61 mL/min). The steady-state piperacillin serum concentration was 83.6 mg/L. Using first-order kinetics, the dose was proportionally reduced to 9 g/day to achieve a target closer to 16 to 64 mg/L (to achieve approximately 1–4x the *P.aeruginosa* isolate MIC). The patient’s mental status improved, and he completed the antimicrobial course without further interruption or intervention.

## Challenges and opportunities

Several factors impede the integration of beta-lactam TDM in OPAT. Sample collection and interpretation are uniquely challenging when beta-lactam TDM is performed in the OPAT setting relative to the hospital. Distinct from other monitorable medications in OPAT (e.g., vancomycin), two beta-lactam serum concentrations are needed to determine an individual pharmacokinetic profile for a patient treated with intermittent infusion beta-lactams (over ≤ 3 hours).^13^

Intravenous access and resource constraints for sample collection (e.g., nursing, phlebotomy) are less reliable in the OPAT setting than in the hospital. Insurance and/or the home health agency staffing may limit the number of home visits in a single day. Patients could present to a facility for blood sampling, but travel and wait time between sample collections (i.e., at least one drug half-life) may be prohibitive. A single sample may be sufficient if drawn as a “true trough” (immediately prior to the next dose) or during continuous infusion therapy, yet continuous infusion therapy is not suitable for all patients due to preference, availability of specialized equipment, and drug stability concerns. Similar to other monitored medications in OPAT, interpretation of resulted beta-lactam levels depends on a detailed understanding of the timing of samples relative to dose administration. Unlike in the hospital, Medication Administration Records (MARs) are rarely available for patients in OPAT. Insurance coverage for beta-lactam TDM in OPAT may also vary. Electronic devices have been successfully used to assess medication adherence; similarly, there may be a future role for digital tracking platforms to record timing of medication administration to assist clinicians interpret concentrations with greater accuracy.^14^ The lack of awareness and education among healthcare providers regarding the benefits of beta-lactam TDM in OPAT may also present a significant barrier to implementation. Many clinicians may not be familiar with the principles of TDM or its relevance to beta-lactam therapy, leading to underutilization. Addressing the knowledge gap through targeted educational initiatives can enhance healthcare providers’ understanding of beta-lactam TDM benefits.

While there are challenges for beta-lactam TDM in the OPAT setting, there are also advantages. In the hospital, beta-lactam dosing and monitoring is managed by a vast array of primary and consultative services, which makes implementation of a TDM program challenging.^13,15^ The small multidisciplinary contingent of infectious diseases experts in OPAT is ideal for TDM training and implementation.^16,17^ These individuals are familiar with adjusting antibiotics based on laboratory values. OPAT clinicians also routinely navigate challenges with outpatient blood sample collection, and have extensive experience interpreting TDM without MARs. Home health staff education on appropriate sample timing can be provided via durable materials or in-services. The majority of beta-lactam assays are performed by reference laboratories, leading to a 1–3 day delay in results reporting. In hospitalized patients, rapidly changing pharmacokinetics, microbiology, and clinical status may render these delayed results clinically uninformative. OPAT treatment plans are usually definitive (i.e., based on resultant microbiology, imaging), average durations are 2–8 weeks, and patients are clinically stable.^16^ These features make the delay in results reporting less consequential in the OPAT setting and the TDM results, when properly obtained, more informative toward the ongoing treatment plan.

Given the unique considerations associated with beta-lactam TDM in the OPAT setting, we propose a general framework of the key factors for OPAT program leadership to consider (Table 1). Key decisions necessary to develop an OPAT beta-lactam TDM program include patient selection, pharmacokinetic/pharmacodynamic targets, and the process map with responsible parties. Legacy workflows of antibiotic TDM in OPAT (e.g., vancomycin) may be adapted for beta-lactams, accounting for unique considerations like the need for multiple samples and send out laboratory testing. Shared decision making between OPAT clinicians and patients is encouraged to discuss the potential benefits of beta-lactam TDM alongside the potential treatment burden (e.g., time, travel, and cost).

**Table 1.** OPAT specific considerations for beta-lactam TDM

Domain	Key questions	OPAT considerations
Patient selection and identification	Which patients would benefit most from beta-lactam TDM?	Consider beta-lactam TDM on a case-by-case basis. Clinical criteria could include individuals at extremes of weight, with unstable or difficult to assess kidney function, with a documented multidrug resistant infection, or with suspected dose-dependent beta-lactam toxicity (e.g., neurotoxicity)
	How will patients be identified and tracked in the EMR?	Patient identification could be proactive, reactive, or both. Proactive methods may include manual screening of OPAT patient lists or via EMR reporting automation for selected patient criteria (body weight, Scr, etc). Reactive methods are in response to emergent concerns about ineffectiveness or about toxicity. Leverage existing OPAT patient tracking methods for other drugs which undergo TDM (e.g., vancomycin)
Sample collection	Will one- or two-point sampling be used?	Two sample monitoring preferred for intermittent infusion beta-lactam therapy One sample monitoring reasonable if a true trough or during continuous infusion therapy
	What are the constraints associated with home monitoring in your environment?	Determine viability of two sample home monitoring with insurance and local home health providers If a non-local patient, discuss viability of travel for sample collection to perform beta-lactam TDM.
Sample processing	Which laboratory will be used to assay beta-lactam concentrations?	Determine viability of developing and validating on-site assays. Most OPAT programs in the United States will use a referral laboratory for beta-lactam monitoring.
	What is the expected turnaround time and how does that affect workflow?	Turnaround time of 1–3 days for most referral laboratories. Select responsible party for ordering and following-up results; given the delayed response these may be different individuals
Results reporting	Is there a process to receive external results in a reliable manner?	Facilitate manual entry of results in the electronic medical record. If not possible, documentation of the result in an OPAT note may suffice. Utilize existing OPAT results reporting strategies for other drugs.
	Does the EMR need to be configured to display beta-lactam TDM results?	Integrate beta-lactam TDM results into existing OPAT reports/dashboards
Clinical interpretation	How will results be interpreted?	Development or implementation of an existing pharmacokinetic calculator to characterize individual beta-lactam pharmacokinetic profile If using population pharmacokinetic model/Bayesian dosing software, choose models based on data from outpatients (rather than hospitalized or critically ill) Recommended regimens may need to be tailored for patient-centeredness in the outpatient setting (e.g., every 6 hour dosing may be infeasible). Continuous infusion or alternative antimicrobials may need to be considered to achieve therapeutic goals.
	Who will be responsible for prescribing changes in beta-lactam doses when necessary?	Clinical pharmacists are best equipped to interpret beta-lactam TDM results. If the pharmacist’s usual OPAT practice involves prescribing of dose changes, this should be extended to changes resulting from beta-lactam TDM.
	How will changes in dose be documented in the EMR?	Develop a templated progress note for OPAT beta-lactam TDM

OPAT: outpatient parenteral antimicrobial therapy, EMR: electronic medical record, TDM: therapeutic drug monitoring

## Conclusion

The scope of precision medicine with beta-lactam TDM should not be limited to hospitalized patients. The potential for beta-lactam TDM in the OPAT setting to improve drug effectiveness and safety is considerable. Thoughtful beta-lactam TDM program development and implementation by a multidisciplinary OPAT team is necessary to overcome clinical and logistical challenges unique to this environment.
